# The impact and distinction of ‘lipid healthy but obese’ and ‘lipid abnormal but not obese’ phenotypes on lumbar disc degeneration in Chinese

**DOI:** 10.1186/s12967-020-02382-0

**Published:** 2020-05-26

**Authors:** Sheng Shi, Zhi Zhou, Jun-Jun Liao, Yue-Hua Yang, Jun-Song Wu, Shuang Zheng, Shi-Sheng He

**Affiliations:** 1 Department of Orthopedics, Shanghai Tenth People’s Hospital, Tongji University, Shanghai, 200072 People’s Republic of China; 2 Spinal Pain Research Institute, Tongji University School of Medicine, Shanghai, 200072 People’s Republic of China; 3 Department of Orthopedics, Fuzhou First People’s Hospital, Nanchang University, Fuzhou, 344000 People’s Republic of China; 4 Department of Orthopedics, The Fifth Affiliated Hospital, Southern Medical University, Guangzhou, 510900 People’s Republic of China; 5 Department of Orthopedics, The First Affiliated Hospital, School of Medicine, Zhejiang University, Hangzhou, 310003 People’s Republic of China; 6 School of Medicine, Shanghai University, Shanghai, 200444 People’s Republic of China

**Keywords:** Hyperlipidemia, Lumbar disc degeneration, Facet joint degeneration, Elevated triglyceride, Abdominal obesity

## Abstract

**Background:**

Lipid abnormality and obesity have been proposed to be associated with lumbar disc degeneration, but little is known about the effect of ‘lipid healthy but obese’ (LH-O) and ‘lipid abnormal but not obese’ (LA-NO) phenotypes on lumbar disc degeneration in Chinese. The study aims to determine the impact and distinction of LH-O and LA-NO phenotypes on lumbar disc degeneration in Chinese, and to identify the association of related factors with risk of lumbar disc degeneration.

**Methods:**

A total of 678 individuals were included with lumbar magnetic resonance imaging, serum lipid levels and anthropometric measurements. Obesity was defined on the basis of body mass index or waist to hip ratio (WHR). Pfirrmann score and Weishaupt’s scale were utilized to assess the degree of disc degeneration and facet joint degeneration.

**Results:**

The incidence of the LH-O and LA-NO phenotypes were 11.4% and 18.1%, respectively. LA-NO phenotype demonstrates a high incidence for disc degeneration (P < 0.05), while LH-O phenotype confers a severe disc degeneration grade (P < 0.05). No statistical difference in the percentage of severe facet joint degeneration grade in each group (P > 0.05). Elevated triglycerides and greater WHR may be the risk factors for lumbar disc degeneration in Chinese.

**Conclusion:**

LH-O and LA-NO phenotypes are common with different status of disc degeneration in Chinese. Elevated triglycerides and abdominal obesity appear to play crucial roles in the development of lumbar disc degeneration.

## Background

Low back pain is a chronic disorder that severely impairs quality of life, and also the leading cause of disability and health burden, worldwide [1]. However, the pathological process of low back pain is complicated and incompletely understood [2], which was ascribed to intervertebral disc degeneration by a variety of investigators [3, 4].

The etiology of disc degeneration has been extensively investigated, but appears to involve biological and biomechanical characteristics such as inflammation, excessive mechanical loading, and an impaired nutrient supply [5–8]. Recently, several reports proposed the causative relationship between lipid abnormality and degenerative disc [9, 10]. Additionally, atherosclerosis induced by hyperlipidemia, may decrease blood flow and nutrient supply to result in the degeneration of a vascularized disc due to the ischemic effect. Moreover, obesity, one of the major public health problems contributing to low back pain, has been implicated in the pathogenesis of disc degeneration due to abnormal and excessive mechanical loading [11, 12]. However, obese patients may not necessarily suffer hyperlipidemia and vice versa [13]. Furthermore, the characteristic relationship between lipid health and weight status has not been convincingly verified for some subtypes of subjects, as ‘lipid healthy but obese’ (LH-O) and ‘lipid abnormal but not obese’ (LA-NO) phenotypes [14, 15]. The former phenotype demonstrates the individuals with a normal lipid profile and obese, while the latter one contains those obese with an abnormal lipid condition. Although the LH-O and LA-NO phenotypes were respectively reported in Europeans, Americans, and other population [16–18], data involving the impact and distinction of LH-O and LA-NO phenotypes on lumbar disc degeneration in Chinese were scarce.

The purposes of this study were to assess the state of disc degeneration in LH-O and LA-NO groups in Chinese, and to determine the association among obesity, hyperlipidemia with lumbar disc degeneration. The phenotypic classification may further enhance our knowledge of the relationship of biological and biomechanical status with disc degeneration.

## Methods

### Study sample

The retrospective analysis was carried out after obtaining the approval of institutional review board of Shanghai Tenth People’s Hospital (SHSY-IEC-4.1/20-45/01). Subjects in the current study were included from January of 2016 to September of 2018 in Shanghai and Hangzhou, two cities in Eastern China, via two approaches. By searching the individuals of health checkups at the authors’ hospitals who had anthropometric measurement, plasma lipid tests, and lumbar magnetic resonance images (MRI). The other approach was from searching the registry in the local departments, the subjects with sufficiently aforementioned assessments were included. Individuals with routine use of lipid-lowing medication, a history of diabetes mellitus, cardiovascular disease, cerebrovascular disease, chronic renal or hepatic failure, cancer, rheumatic diseases, hyperthyroidism and hypothyroidism, and those unsuitable for analysis, were excluded from the present study.

### Data collection

Waist circumference (WC) was considered as the circumference midpoint between the lowest rib and the iliac crest, through the umbilicus. Hip circumference was documented as the widest gluteal circumference. Circumference measurements were with standard methods. Circumferences were measured by a qualified physician at listed locations three times using body tape measure. The results were given in centimeters with 0.5 cm accuracy. Then the mean values were recorded by another physician. Waist to hip ratio (WHR) was defined as WC (cm) divided by hip circumference (cm). Body mass index (BMI) was calculated as the body weight (kg) divided by the body height squared (m^2^). Obesity was recorded on the basis of BMI or WHR. Individuals with BMI ≥ 28 kg/m^2^ according to Chinese BMI classification standard [19] or abdominal obesity according to high WHR in the previous report [20]. High WHR was considered as ≥ 0.85 in women and 0.9 in men [21, 22].

After a more than 8-h overnight fasting, plasma sample from each subject was measured for the lipids levels (Hitachi 7020, Hitachi Co. Tokyo, Japan). Lipid levels were recorded utilizing fully automatic biochemistry analyzer. Lipid abnormality was considered when triglyceride (TG) levels were ≥ 1.7 mmol/L and/or total cholesterol (TC) levels ≥ 5.2 mmol/L and/or high density lipoprotein-cholesterol (HDL-C) levels < 1.04 mmol/L and/or low density lipoprotein-cholesterol (LDL-C) levels ≥ 3.5 mmol/L [23–26].

### Phenotype assessment

Individuals in the current investigation were separated into four groups as follows: lipid healthy and non-obese (LH-NO), lipid healthy and obese (LH-O), lipid abnormal and non-obese (LA-NO) and lipid abnormal and obese (LA-O). LH-NO was shown as having healthy lipid profile and no obesity. LH-O was shown as having obesity but healthy lipid profile. LA-NO was shown as having lipid abnormality but no obesity. LA-O was shown as having lipid abnormality and obesity.

### Radiological measurement

The MRI of lumbar spine was scanned through a standard protocol by a 1.5 Tesla scanner (GE, Signa HDxt, USA). On T2-weighted sagittal images, the degree of disc degeneration from whole lumbar segments (L1/2 segment to L5/S1 segment) was assessed by Pfirrmann score [27]. This validated score is generally adapted in clinical study and graded by the structure, signal intensity, distinction between nucleus and annulus, and height of disc. Weishaupt’s scale [28], on the basis of the narrowing space, hypertrophy, osteophytes, and bone erosions of the zygapophysial joints, was utilized to evaluate the facet joint degeneration. All the images were measured and assessed independently by a radiologist and an orthopaedic surgeon. If there was the discrepancy in the radiological assessment between radiologist and orthopedic surgeon, the radiologist made the final decision after consulting with orthopedic surgeon.

### Statistical analysis

All statistical analyses were conducted utilizing SPSS Version 22.0 (SPSS Inc., Chicago, IL, USA). Normality of data was identified by applying the Kolmogorov–Smirnov test. Data were expressed as mean ± standard deviation for normally distributed continuous variables or as median (interquartile range 25–75%) for skewed variables. Group based differences were compared by analysis of variance (ANOVA) following SNK post hoc pairwise comparison for normality distributed data or Kruskal–Wallis H test following Mann–Whitney U test for skewed data. Intra-class correlation coefficient (ICC) was utilized to determine inter-observer reliability for radiological measurements with a higher value indicating better reliability. An ICC value from 0.40 to 0.59 was considered as fair, 0.60 to 0.74 as good, and greater than 0.75 as excellent [29]. Multivariate logistic regression analysis including all significant variables in univariate analysis was performed to determine the significant risk factors for development of disc degeneration. A P value < 0.05 was considered significant.

## Results

### Basic conditions of individuals

In total, 678 individuals with 3390 lumbar discs were included in the current study. The basic conditions of the individuals in each group are presented in Table 1. Of all individuals included in the current study, 14.9, 11.4, 18.1, and 55.6% were divided into LH-NO, LH-O, LA-NO, and LA-O, respectively (Table 1). There were no differences in the above proportion in each group between men and women. According to the definition, indices of obesity (including BMI and WHR) were higher in LH-O than in LH-NO groups (P < 0.05), also higher in LA-NO than in LH-NO groups (P < 0.05). However, no statistical difference in obesity between LH-O and LA-O groups was documented. Moreover, LH-O individuals also had higher TG, low-density cholesterol (LDL-C) and lower HDL-C levels than LH-NO individuals (all P < 0.05). No difference was shown in TC levels between these two groups (P > 0.05).

**Table 1:** Baseline characteristics of subjects

	LH-NO	LH-O	LA-NO	LA-O
Number	101	77	123	377
Gender (M/F)	48/53	32/45	64/59	178/199
Age (year)	48.2 ± 8.3	47.0 ± 10.4	48.8 ± 7.5	48.2 ± 9.2
BMI (kg/m^2^)	22.01 ± 2.74	24.15 ± 3.64^a^	22.61 ± 3.08^b^	24.88 ± 3.30^a,c^
WHR	0.82 (0.80, 0.84)	0.91 (0.89, 0.95)^a^	0.82 (0.78, 0.84)^b^	0.91 (0.89, 0.94)^a,c^
TG (mmol/L)	0.90 (0.72, 1.14)	1.08 (0.81, 1.48)	1.66 (1.06, 2.01)^a,b^	2.25 (1.61, 3.10)^a,b,c^
TC (mmol/L)	4.59 (4.22, 4.79)	4.71 (4.30, 4.96)	5.40 (5.09, 5.84)^a,b^	5.44 (5.12, 6.18)^a,b^
HDL-C (mmol/L)	1.47 (1.29, 1.76)	1.34 (1.26, 1.51)^a^	1.45 (1.13, 1.78)^b^	1.23 (1.06, 1.38)^a,b,c^
LDL-C (mmol/L)	2.60 (2.12, 2.79)	2.76 (2.37, 3.02)	3.27 (2.84, 3.78)^a,b^	3.38 (3.02, 3.83)^a,b^
Subject percentage (%)	20.8	31.2	25.2	39.0
DD percentage (%)	4.2	6.5	8.3	13.5

Data were expressed as mean ± standard deviation for normal distribution (age and BMI) and as median (interquartile range 25–75%) for skewed variables (WHR, TG, TC, HDL-C, and LDL-C)

*BMI* body mass index, *WHR* waist to hip ratio, *TG* triglyceride, *TC* total cholesterol, *HDL-C* high-density lipoprotein cholesterol, *LDL-C* low-density lipoprotein cholesterol, *LH-NO* lipid healthy and non-abdominal obese, *LH-O* lipid healthy and abdominal obese, *LA-NO* lipid abnormal and non-abdominal obese, *LA-O* lipid abnormal and abdominal obese, *DD* degenerated disc

^a^Means P < 0.05 versus LH-NO

^b^Means P < 0.05 versus LH-O

^c^Means P < 0.05 versus LA-NO

The results of the ICC analysis for disc degeneration and facet joint degeneration measurements mainly indicated good to excellent agreement. The reliability statistics for disc degeneration grade was 0.92 for intraobserver reliability and 0.86 for interobserver reliability. The reliability statistics for facet joint degeneration grade was 0.89 for intraobserver reliability and 0.73 for interobserver reliability.

### Comparison of the degeneration status in individuals from each group

**Fig. 1:**
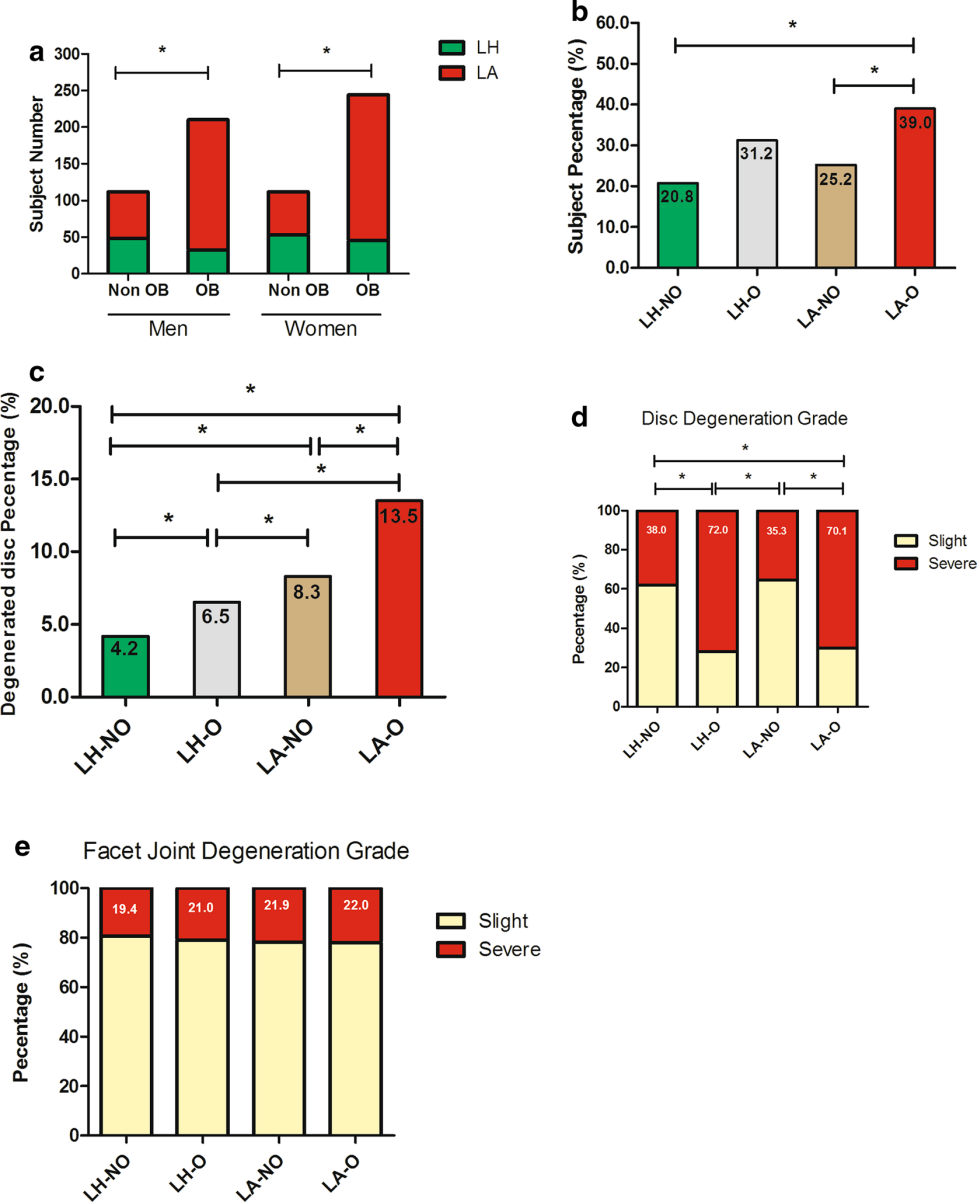
Comparison of the degeneration status of lumbar disc and facet joint in individuals from each group. **a** Lipid abnormal (LA) over lipid healthy (LH) ratio; **b** subject percentage with disc degeneration; **c** degenerated disc percentage; **d** severe over slight ratio in the disc degeneration grade; **e** severe over slight ratio in the facet joint degeneration grade. *OB* obesity, *LH-NO* lipid healthy but no obese, *LH-O* lipid healthy but obese, *LA-NO* lipid abnormal but no obese, *LA–O* lipid abnormal and obese. *Means P < 0.05 versus indicated groups

Of note, the lipid abnormal over lipid healthy ratio was higher in obesity than in non-obesity both for men and women (Fig. 1a, P < 0.01). Individuals from the LA-O group had the highest percentage (39.0 and 13.5% for individual number and disc number, respectively) of disc degeneration (Fig. 1b, c). However, the percentage of individuals with disc degeneration in LA-O group is similar with that in LH-O group (P > 0.05). As for the involved number of degenerated disc, the ratio of numbers with disc degeneration increased across each groups from LH-NO, LH-O, LA-NO, and LA-O groups (P for trend < 0.05). Furthermore, the ratio of disc numbers with high Pfirrmann score, a severe disc degeneration grade (Grade 4 and Grade 5), was similar between LH-O and LA-O groups. It was statistically higher in LH-O group than that in both LA-NO group (P < 0.05) and LH-NO (P < 0.05) (Fig. 1d). For all subjects included in the four groups, there was no statistical difference in the percentage in the severe facet joint degeneration (Grade 3 and Grade 4) in each group (P > 0.05) (Fig. 1e).

### Multivariate logistic regression for the risk of intervertebral disc degeneration

Greater WHR, elevated TG and decreased HDL-C were associated with disc degeneration (P < 0.01 for both, Table 2). As indicated in Table 3, the odds ratio (OR) for having disc degeneration were an elevated TG, and WHR. Elevated TG was associated with disc degeneration (OR = 1.243–1.629, P < 0.01). Increased WHR was also statistically associated with disc degeneration (OR = 1.052–1.121, P < 0.01).

**Table 2:** Correlation analysis among multivariates and disc degeneration in different groups and the whole people

Variables	LH-NO		LH-O		LA-NO		LA-O		Whole	
	Disc degeneration		Disc degeneration		Disc degeneration		Disc degeneration		Disc degeneration	
	β	P value	β	P value	β	P value	β	P value	β	P value
Age	0.076	0.451	− 0.076	0.513	0.069	0.447	0.057	0.267	0.041	0.286
Gender	0.243	0.014	− 0.172	0.134	0.042	0.642	− 0.006	0.900	0.018	0.635
BMI	0.084	0.406	0.284	0.012	0.200	0.027	0.116	0.024	0.189	< 0.001
WHR	0.045	0.654	0.449	< 0.001	0.432	< 0.001	0.158	< 0.001	0.256	< 0.001
TG	− 0.027	0.790	0.345	0.002	0.511	< 0.001	0.198	< 0.001	0.270	< 0.001
TC	− 0.048	0.630	0.087	0.454	0.071	0.438	0.084	0.105	0.117	0.002
HDL-C	0.040	0.694	− 0.370	0.001	− 0.210	0.020	− 0.056	0.274	− 0.161	< 0.001
LDL-C	− 0.198	0.057	− 0.238	0.037	0.095	0.295	0.043	0.400	0.058	0.129

*BMI* body mass index, *WHR* waist to hip ratio, *TG* triglyceride, *TC* total cholesterol, *HDL-C* high density lipoprotein-cholesterol, *LDL-C* low density lipoprotein-cholesterol

**Table 3:** Multivariate logistic regression of multiple covariates and the risk of disc degeneration in different groups and the whole people

Groups	Variables	Risk of disc degeneration	
		OR (95% CIs)	P value
LH-NO	Gender (F/M)	3.719 (1.243, 11.129)	0.019
LH-O	TG (mmol/L)	8.485 (1.513, 47.594)	0.015
	WHR (%)	1.294 (1.108, 1.512)	0.001
LA-NO	TG (mmol/L)	3.021 (1.635, 5.582)	< 0.001
	WHR (%)	1.350 (1.142, 1.595)	< 0.001
LA-O	TG (mmol/L)	1.377 (1.167, 1.625)	< 0.001
	WHR (%)	1.101 (1.027, 1.169)	0.002
Whole	TG (mmol/L)	1.423 (1.243, 1.629)	< 0.001
	WHR (%)	1.086 (1.052, 1.121)	< 0.001

Odds ratio (OR) and confidence interval (CI) were determined by stepwise logistic regression after adjusted for age, BMI, TC, HDL-C, LDL-C

*TG* triglyceride, *WHR* waist to hip ratio

## Discussion

In the current study, we revealed that certain subtypes of obesity with different lipid status are presented in the Chinese population. The incidence of the LH-O and LA-NO individuals in the Eastern China were 11.4% and 18.1%, respectively. Furthermore, individuals from the LA-NO group had a higher segment percentage of disc degeneration, whereas a severe disc degeneration grade was detected statistically for subjects with LH-O. Elevated TG and abdominal obesity may be the risk factors for lumbar disc degeneration in Chinese. To our best knowledge, this is the first study to identify LH-O and LA-NO subjects, and associate those with disc degeneration by utilizing MRI in a large sample of Chinese adults.

It is noted that approximately 65% of the subjects in this study were obese. First, the average age of the individuals included in this study was 50 years old, often concomitant with obesity. Second, the definition of obesity in this study is based on BMI or WHR, which means the general or abdominal obesity also belongs to obesity. However, Asians have higher body fat content than Western people with the same BMI and tend to suffer central obesity. Last, a recent literature from the China Health and Nutrition Survey also revealed the age-adjusted incidence rate of abdominal obesity was 43.15%, and it increased year by year [30].

In the present study, the differences between LA-NO and LH-O phenotypes with disc degeneration differ in the segment percentage and degenerated grade. Indeed, it is proposed that the adipose tissue and/or dyslipidemia may elicit pro-inflammatory responses and promote apoptosis through the secretion of pro-inflammatory cytokines, which exert a catabolic effect on disc cells, and then result in disc degeneration. Generally, the intervertebral disc is an avascular structure that mainly relies on diffusion through the endplate for nutrition, hence disc structures with precarious nutrient supply might gradually degenerate as a consequence of failure of nutrient supply to disc cells due to atherosclerosis caused by hyperlipidemia. From the opposite side, another study postulated that the utilization of statins, lipid-lowering drug, may retard the pathological process of disc degeneration via improving the blood supply and decreasing the inflammation [31]. Admittedly, the subjects included in this cross-sectional study were not receiving lipid-lowering medications, which can not reveal the potential effects of statins in this study. Therefore, the difference in the percentage of disc degeneration between LA-NO and LH-O phenotypes may be partly explained by the nutrient supply insufficiency systematically affecting metabolism of the disc cells besides inflammatory factors.

Apart from the effect of systemic pro-inflammatory factors produced persistently by obese adipose tissue, it was recently shown that obesity may alter the lumbar biomechanics and then accelerate the disc degeneration [32]. Another recent study speculated that mobile segments of lumbar intervertebral discs were easily mediated by mechanical stress and the effect of metabolic factors on the discs may be covered concurrently [33]. Similarly, Samartzis et al. [34] also reported an elevated BMI was significantly associated with increased severity of disc degeneration and obese individuals had greater severity of disc degeneration than normal individuals. Additionally, a meta-analysis involving the association between obesity and lumbar disc degeneration demonstrated that overweight was significantly associated with degeneration [35]. Teraguchi et al. [33, 36] also revealed that metabolic factors may influence the incidence of disc degeneration in the upper and lower lumbar spine, while mechanical stress easily affected the disc in the lower lumbar spine. Consistent with the aforementioned studies, our results also indicated the altered biomechanics induced by obesity may affect the degenerative change of mobile discs easily and strongly, but lipid abnormality may affect the process systematically and weakly.

No significant difference in the percentage of facet joint degeneration grade was observed in each group. Unlike the disc allowing for motion in all directions, the facet joints are usually limited by its shape, orientation, and ligamentous capsules [37], which is considered to defend the discs against excessive sheer or torsional stiffness. Moreover, intervertebral discs, the crucial elements of anterior column in the vertebral column, are assumed to transfer the major physical or external loading (about 75–97%) [38]. Accordingly, the facet joints may occasionally suffer excessive stress and degenerative pathological process, when the disc degenerative changes advanced into unstable phase from dysfunction phase. Additionally, current investigation also indicated that facet joint degeneration was usually preceded by disc degeneration, which appeared probably at the segment with severe disc degeneration [39]. Thus, we thought the effect of lipid and obesity on the joint facet degeneration can ultimately be found in a longitudinal study with a larger sample.

Among these hyperlipidemia parameters, elevated TG was a significant risk factor for disc degeneration in our study. Furthermore, hypertriglyceridemia was also indicated to lead to disc degeneration via catabolic degeneration and apoptosis in disc cells by several reports [10, 40–43]. Admittedly, abdominal obesity, another risk factor in this study, was thought to be associated with disc degeneration. Available reports increasingly indicate that such subjects, based on visceral fat accumulation with insulin resistance, are predisposed to hypertriglyceridemia, atherosclerosis, type 2 diabetes mellitus, and premature coronary heart disease. Different from healthy obese phenotype described as ‘fat and fit’, ectopic fat visceral deposition may lead to mechanical loading and/or local leptin in the disc structure, and eventually disc degeneration [15, 44]. Recently, Segar et al. [5] also pointed out that leptin can result in a degenerative and inflammatory cascade in the intervertebral disc.

Some limitations could not be avoided in this study. Undeniably, selection bias may exist in this retrospective analysis. Moreover, no information about whether disc degeneration correlates with low back pain may result in a limitation to this study. Despite a relatively large-sample size, the subjects included in this study may not represent the general population because they were included from only two local cities and restricted to those available for MRI evaluation. As the present study was a cross-sectional study without a longitudinal follow-up, we could not confirm temporality or the effects of lipid and obesity status on the development of degeneration in the discs and facet joints. No precise confounding factors about the physical activity, medication, diabetes mellitus and lifestyle state such as smoking was investigated in the analysis, which may also be deemed to be related to disc degeneration.

## Conclusion

In summary, LH-O and LA-NO phenotypes are common in Chinese. LA-NO phenotype demonstrates a high incidence for disc degeneration, while LH-O phenotype confers a severe disc degeneration grade. Elevated TG and abdominal obesity may be the risk factors for lumbar disc degeneration in Chinese. However, the precise mechanisms behind the phenomenon are not clearly clarified. Differentiating LH-O and LA-NO from other phenotypes is essential for guiding the treatment strategies for patients with disc degeneration.

## Data Availability

The datasets used and/or analyzed during the current study are available from the corresponding author on reasonable request.
